# A New Insight on Atherosclerosis Mechanism and Lipid-Lowering Drugs

**DOI:** 10.31083/RCM25321

**Published:** 2025-03-05

**Authors:** Penghui Li, Wei Jiang

**Affiliations:** ^1^Binhai New Area Hospital of TCM, 300000 Tianjin, China; ^2^First Teaching Hospital of Tianjin University of Traditional Chinese Medicine, 300000 Tianjin, China

**Keywords:** atherosclerosis, inflammation, intestinal microbiota, extracellular vesicles, programmed cell death, non coding RNA, lipid-lowering therapy, lipid-lowering drugs

## Abstract

Atherosclerosis (AS) is a chronic vascular disease primarily affecting large and medium-sized arteries, involving complex pathological mechanisms such as inflammatory responses, lipid metabolism disorders and vascular plaque formation. In recent years, several emerging research hotspots have appeared in the field of atherosclerosis, including gut microbiota, pyroptosis, ferroptosis, autophagy, cuproptosis, exosomes and non-coding RNA. Traditional lipid-lowering drugs play a crucial role in the treatment of AS but are not able to significantly reverse the pathological changes. This article aims to summarize the latest research progress in the pathogenesis of AS and the diagnosis and treatment of the disease by comprehensively analyzing relevant literature mainly from the past five years. Additionally, the mechanisms of action and research advances of statins, cholesterol absorption inhibitors, fibrates and novel lipid-lowering drugs are reviewed to provide new insights into the diagnosis and treatment of AS.

## 1. Introduction

Atherosclerosis (AS) serves as the common pathological foundation for various cardiovascular and cerebrovascular diseases, involving macrophages, endothelial cells (ECs), smooth muscle cells, lymphocytes and intercellular signaling factors [1, 2]. The interplay between these cells and cytokines collectively drives AS progression. Its main pathological features include lipid deposition, immune cell infiltration and inflammatory responses, which can go through stages of fatty streaks, fibrous plaques and atheromatous plaques that even cause intraplaque hemorrhage, rupture and thrombus formation [3, 4, 5]. The complexity of AS arises from the interplay of multiple factors and mechanisms, posing significant challenges for the diagnosis and treatment of AS-related diseases. Therefore, in-depth research into the pathogenesis of AS is of paramount importance for exploring novel diagnostic and therapeutic strategies. In recent years, numerous international scholars have investigated new mechanisms of AS formation and progression in areas such as inflammation, gut microbiota, pyroptosis, ferroptosis, autophagy, cuproptosis, exosomes and non-coding RNA [6, 7, 8, 9, 10]. This article mainly reviews these aspects, aiming to provide new insights into the mechanistic research and intervention strategies for AS. Due to space limitations, this review did not cover the extensive body of research and review reports on classic lipid metabolism disorders and their effects on the onset and progression of AS and its interventions.

## 2. Inflammation and AS

AS is a chronic, progressive vascular inflammatory disease, typically characterized by the accumulation of lipids and inflammatory responses within the arterial walls [11]. Numerous studies have demonstrated that inflammation plays a crucial role in the formation and progression of AS lesions [11]. In addition to its direct impact on the advancement of AS-related diseases, inflammation also intertwines with various pathogenic factors such as lipid accumulation and oxidative stress, adding to the complexity of lesion development [12]. Despite the availability of several inflammation-targeted therapies for AS, their efficacy remains limited, prompting researchers to delve deeper into the relationship between inflammation and AS.

Previous studies have identified several inflammatory markers such as C-reactive protein (CRP), interleukin-6 (IL-6), adhesion molecules and copeptin (a C-terminal peptide of the precursor to vasopressin), which exhibit strong predictive capabilities for AS [12, 13, 14]. Recently, the complement system within the immune response has also been reported to regulate AS progression [13, 14, 15, 16, 17, 18, 19, 20]. In an AS model induced by a high-fat diet in low-density lipoprotein receptor (*LDLR*)^-⁣/-^ mice, macrophages derived from monocytes can activate complement C3 through the expression of complement factor H (CFH), thereby promoting inflammation progression. The absence of CFH effectively inhibits C3-dependent plaque necrosis, suggesting that CFH plays a critical role in the inflammatory progression of AS by regulating C3 levels within macrophages [13]. This finding not only elucidates the relationship between the complement system and AS but also provides new directions for subsequent research. Moreover, new inflammation-related genes have been discovered to regulate AS progression. Myeloid-derived growth factor (MYDGF) [14], a secreted protein produced by inflammatory cells including bone marrow-derived monocytes and macrophages, has been shown to promote the synthesis and secretion of glucagon-like peptide-1 by intestinal L cells to alleviate inflammatory responses on vascular ECs [15, 16]. Recent studies have found that serum MYDGF levels are significantly decreased in patients with lower limb AS and in AS mice, and the specific deletion of MYDGF in inflammatory cells exacerbates AS [14, 17]. Bone marrow transplantation to restore or overexpress MYDGF in inflammatory cells can ameliorate the effect of inhibiting the progression of AS [17]. This suggests that key gene editing in inflammatory cells through bone marrow transplantation could be a novel intervention strategy for AS. Additionally, in apolipoprotein E (*ApoE*)^-⁣/-^ mice, the natriuretic peptide receptor C (*NPRC*), a gene significantly associated with coronary artery disease, is upregulated in AS plaques [18]. Its deficiency can alleviate AS by inhibiting inflammation, oxidative stress and apoptosis, indicating that targeting NPRC may represent a new strategy for AS intervention [18]. Other potential targets for addressing inflammation in AS include doublecortin-like kinase 1 (DCLK1) [19] and zinc finger protein family transcription factor 6 (Gata6) [20].

In summary, these studies suggested that in addition to traditional inflammatory markers, many new genes can also affect the progression of AS by regulating inflammation. This also prompts us to consider that some non-classical genes that can regulate AS inflammation should also be included in the evaluation of AS inflammation. Therefore, in-depth exploration of the mechanisms of inflammatory responses and new intervention strategies are crucial for improved cardiovascular health.

## 3. Intestinal Microbiota and AS

Intestinal microbiota refers to a collection of various microbial communities that reside in the host’s gut and coexist with the host. Previous studies have found that dysbiosis of the gut microbiota is closely related to gastrointestinal diseases, while recent studies have also shown that the dysbiosis can affect the progression of AS [21, 22, 23, 24, 25, 26].

Existing reports have indicated that patients with AS exhibit higher abundances of *Enterobacteriaceae* and *Streptococcus* in their gut microbiota, and these genera are involved in the metabolism and transport of several key cardiovascular-protective molecules [21]. This suggests that dysbiosis of gut microbiota is closely related to AS, and the mechanism behind it is worth exploring further. As previously mentioned, the inflammatory response is fundamental to the development and progression of AS. In patients with coronary artery disease, the abundances of *Coriobacteriaceae* and *Ruminococcaceae* were significantly reduced, with some members of these families producing butyrate, which has anti-inflammatory properties [22]. Transplanting pro-inflammatory gut microbiota from *Caspase-1*^-⁣/-^ mice into *LDLR*^-⁣/-^ mice resulted in elevated plasma pro-inflammatory cytokine levels and accelerated AS formation [23]. Supplementing with *Bacteroides* fragilis could also exacerbate inflammation to promote AS progression [24]. Additionally, gut microbiota dysbiosis can lead to the production of endotoxins that enter the portal vein and activate the NLR Family, pyrin domain containing protein 3 (NLRP3) inflammasome [25]. Administration of peanut skin extract (PSE) has been found to modulate gut microbiota composition and reduce the levels of pro-inflammatory cytokines tumor necrosis factor-α (TNF-α) and IL-6, thereby slowing AS progression [26]. These findings suggest that inflammation induced by gut microbiota dysbiosis may be a critical mechanism for AS.

Hypercholesterolemia is another important factor contributing to AS. The gut microbiota dysbiosis induced by antibiotics can promote developing hypercholesterolemia leading to be associated cardiovascular diseases (CVDs) [27]. Administration of *Lactobacillus* and *Bifidobacterium* could reduce cholesterol levels [28]. Niemann-Pick C1 like 1 (NPC1L1), a protein essential for cholesterol absorption, was upregulated in AS models induced by high-fat diets in *ApoE*^-⁣/-^ mice, and *Lactobacillus*_UCG-008 and *Roseburia* species have been found to increase its expression [29]. This suggests that maintaining gut microbiota homeostasis has significant therapeutic value for AS caused by hypercholesterolemia. Further research has found that gut microbiota metabolites also participate in AS progression. For instance, trimethylamine-N-oxide (TMAO) can stimulate inflammation by increasing cellular reactive oxygen species (ROS) levels, thereby promoting AS [30], whereas indole-3-propionic acid can inhibit AS progression [31]. These conclusions indicated the importance of balancing gut microbiota metabolites.

Current research on the relationship between gut microbiota and AS has become a focal point, with many questions remaining to be explored and answered. For example, is gut microbiota dysbiosis a direct cause of AS, or does it merely share common influencing factors with the formation and progression of the disease? Which specific gut microbiota species are definitively correlated with the development of AS, and what are the mechanisms involved? Furthermore, some studies have discovered that altering a person’s diet using probiotics or administering antibiotics can adjust the composition of gut microbiota, thereby exerting antagonistic effects on AS [30, 31, 32]. However, the optimal strategies for their adjustment and safety require more in-depth research.

## 4. Cell Death and AS

Cell death is a normal biological phenomenon which can occur by various mechanisms, resulting in different forms of death including apoptosis, pyroptosis, autophagy, ferroptosis and cuproptosis. However, the roles of these different forms of cell death in disease processes may differ. The presence of these forms of cell death have been identified in AS plaques, but the specific mechanisms or whether these cell death modes are regulated by a common signaling pathway remain unclear. Exploring these new mechanisms in AS may provide new perspectives for the intervention of AS mechanisms.

### 4.1 Pyroptosis and AS

Pyroptosis is a novel form of programmed cell death dependent on inflammatory caspases and the gasdermin (GSDM) protein family [33]. Recent studies have indicated that pyroptosis can occur in various cells involved in AS such as ECs, macrophages and vascular smooth muscle cells (vSMCs), thereby influencing disease progression [30, 31, 32]. As a barrier of the vascular wall, ECs are crucial for the development of AS when their function is impaired. A recent study reported that high expression of GTPase activating protein 1 (IQGAP1), a critical scaffolding protein regulating mitochondrial function, can induce EC pyroptosis, thereby accelerating AS formation [34]. Treatment with Tongxinluo (a type of Chinese herbal medicine) can reduce EC pyroptosis and plaque area in an AS mouse model, thereby slowing AS progression [35]. These results suggested that regulating EC pyroptosis may be a novel strategy against AS. Macrophages are key effector cells in AS progression. Hyperhomocysteinemia, an independent risk factor for AS, can promote macrophage pyroptosis by inducing endoplasmic reticulum stress and disrupting calcium homeostasis, thus accelerating AS progression [36]. Apigenin can delay AS progression by inhibiting macrophage pyroptosis, indicating that macrophage pyroptosis plays a significant role in the progression of AS [37]. Additionally, it has been reported that in high-fat diet-fed *ApoE*^-⁣/-^ mice, the expression of the olfactory receptor 2 (OLFR2) in macrophages is increased, which can exacerbate AS progression by activating NLRP3 and downstream caspase-1 to release IL-1β, which was also observed in human macrophages [38]. vSMCs are important components in AS, and recent research has found that human cytomegalovirus can promote vSMC proliferation and invasion, and inhibit vSMC pyroptosis, potentially promoting AS progression [39].

Although there has been some progress in understanding the relationship between pyroptosis and AS, the extent of its influence and its underlying mechanisms in AS development remain unclear. Whether there are interactions with other forms of cell death and their effects on AS lesion formation have not been well elucidated to date.

### 4.2 Ferroptosis and AS

Ferroptosis is also one of the novel programmed cell death caused by the accumulation of iron-dependent lipid peroxides [40]. Mouse models with ferroportin (FPN) mutations have been shown to have elevated non-transferrin-bound serum iron (NTBI) and subsequent iron overload, a significant increase in AS plaque area and lipid peroxidation [41]. In AS models induced by a high-fat diet in *ApoE*^-⁣/-^ mice, the iron content in the serum and aortic tissue increased significantly, and the ferroptosis inhibitor Fer-1 reduced iron accumulation with decreased solute carrier family 7 member 11 (SLC7A11) and glutathione peroxidase 4 (GPX4) levels, eventually alleviating AS [42]. These data support the involvement of ferroptosis in AS progression. However, the specific mechanisms by which ferroptosis contributes to AS progression remain to be explored.

The Xc- system, composed of the light chain subunit SLC7A11 and the heavy chain subunit solute carrier family 3 member 2 (SLC3A2), is a crucial transporter of cystine that is a rate-limiting precursor for intracellular glutathione (GSH) synthesis. When the Xc- system is imbalanced, cells lose their antioxidant capacity resulting in lipid peroxidation and subsequent cell death [43]. A study has found that in the carotid plaques of smokers, macrophages exhibit significant decreases in FPN and SLC7A11 with an upregulation of hepcidin [42]. High-fat diet-fed *ApoE*^-⁣/-^ mice showed significant increases in AS plaque and necrotic core areas, along with macrophages in plaques displaying ferroptosis, possibly due to dysregulation of the FPN/SLC7A11 axis [44]. Additionally, overexpression of SLC7A11 in macrophages *in vitro* can mitigate tar-induced increases in ferrous ion accumulation, lipid peroxidation and GSH depletion [44]. This suggests that SLC7A11 may be a novel target for intervention in smoking-induced AS. GPX4 is an important regulator of iron that can reduce lipid peroxides to harmless lipid alcohols by utilizing GSH, thereby protecting cells from lipid peroxidation toxicity [45]. Overexpression of GPX4 can reduce lipid peroxidation and AS damage in high-fat diet-fed *ApoE*^-⁣/-^ mice, while a GPX4 knockout can promote foam cell formation in macrophages and accelerate intracellular cholesterol accumulation [46, 47], indicating that GPX4 may be a key factor in AS formation. Additionally, studies on ferroptosis-related molecules such as acyl-coenzyme A (CoA) synthetase long chain family member 4 (ACSL4) and nuclear factor erythroid 2-related factor 2 (Nrf2) have provided new insights into the mechanisms of AS formation [48, 49].

Beyond the classical pathways, non-classical pathways such as non-coding RNAs and vitamin D receptors can also regulate ferroptosis and participate in AS progression [50, 51]. Research on the relationship between ferroptosis and AS provided potential targets for new intervention strategies. However, for the distribution of ferroptosis within the arterial wall in AS, its interactions with other cell death mechanisms and how it influences plaque formation and instability warrant further investigation.

### 4.3 Autophagy and AS

Autophagy is the form of programmed cell death discovered by Clark SL in 1957 [52]. In mammalian cells, there are three subtypes: macroautophagy, microautophagy and chaperone-mediated autophagy (CMA). These subtypes primarily handle the degradation of damaged or excess cytoplasmic proteins and organelles to maintain cellular homeostasis. In AS plaques, autophagy-triggering factors such as ROS, low-density lipoprotein (LDL) and inflammatory mediators are prevalent [53]. Earlier studies suggested that autophagy mainly promotes AS progression [54, 55], while more recent research has revealed that enhancing autophagy can slow AS progression, particularly focusing on macrophages, thereby providing a theoretical basis for targeting macrophage autophagy as an AS intervention.

Recent reports indicated that treatment with the autophagy inhibitor chloroquine exacerbated AS lesions in mice, whereas, treatment with calycosin promoted autophagy and inhibited inflammation *via* the kruppel like factor 2 (KLF2)-mixed lineage kinase domain-like (MLKL) signaling pathway, thereby delaying AS progression in mice [56], which suggested that moderate autophagy can exert anti-AS effects. Autophagy related protein 14 (ATG14) is a crucial regulator of the fusion between autophagosomes and lysosomes. Impaired autophagic function and downregulation of ATG14 have been observed in AS plaques of both humans and mice [57]. Upregulation of ATG14 can reverse impaired autophagic function in plaque macrophages by inhibiting inflammation, thereby slowing AS progression [57]. Oxidized LDL (OX-LDL) is a key factor in AS, recognized and phagocytosed by scavenger receptors on subendothelial macrophages, leading to lipid overload and triggering an inflammatory response in the AS process. A study has found that OX-LDL treatment impaired macrophage autophagic function and induced inflammation, whereas phosphatidylethanolamine (PE) treatment could induce autophagy and reduce inflammasome formation to alleviate inflammation [58]. These findings suggested that promoting macrophage autophagy in AS to reduce inflammation may be a new strategy for AS intervention.

It should be noted that autophagy has a dual role in AS progression. Appropriate autophagy in the early stages of AS can delay disease progression, whereas excessive autophagy in the late stages may accelerate disease deterioration [59, 60]. However, methods for quantifying the degree of autophagy, the correlation between autophagy levels and the intensity of stimuli and the use of data modeling to assess the relationship between autophagy and AS require further in-depth research.

### 4.4 Copper Death and AS

Cuproptosis is a form of metal ion-dependent cell death [61]. Recent research suggests that the homeostasis imbalance of copper ions is closely related to the development of AS [62, 63]. Kuzan A *et al*. [62] found that copper content in aortic tissue from autopsy specimens was decreased, indicating a negative correlation with the age and severity of AS. In an AS rabbit model induced by a high-cholesterol diet, a decrease in copper content was observed in the vascular walls, heart, liver and other organs, with a negative correlation between aortic copper content and AS severity, however, higher copper levels were found in the serum and kidneys [63].

Additionally, molecules regulating cuproptosis also participate in AS progression. Copper transporter protein alpha chain protein (ATP7A), a copper transport protein, releasing intracellular copper to the extracellular space to promote LDL oxidation. In the *LDLR*^-⁣/-^ mouse AS model, macrophage ATP7A expression was upregulated, and interfering with its expression could reduce cell-mediated LDL oxidation in AS [64]. Supplementing the diet with appropriate copper levels can reduce AS lesion formation [65]. This suggested that copper deficiency can lead to AS. However, it is noteworthy that high dietary copper supplementation in high-cholesterol-fed rabbits led to high copper concentrations in the aorta and increased AS susceptibility [66, 67]. Additionally, molecules related to cuproptosis such as ferredoxin 1 (FDX1) and SLC31A1 (high affinity copper uptake protein 1, solute carrier family 31, member 1) were significantly upregulated in AS plaques, and using copper chelators and copper ion carrier inhibitors could delay AS progression in mice [68, 69]. This indicated that both copper accumulation and increased cuproptosis can lead to AS. However, the dynamic changes in copper ion content during AS progression and the mechanisms by which cuproptosis regulates AS development require further exploration [70, 71, 72].

## 5. Exosomes and AS

Exosomes are lipid bilayer vesicles derived from various cells that can carry a variety of bioactive molecules such as proteins, RNA, DNA, glycans and metabolites [73, 74]. Many studies have focused on the non-coding RNAs carried by exosomes, which are involved in various biological processes of recipient cells during AS such as apoptosis, proliferation, migration and inflammation [73, 74, 75]. However, the biological functions of exosomes vary significantly depending on their cargo. For instance, macrophages treated with ox-LDL can produce exosomes loaded with either long non-coding RNA (lncRNA) GAS5 (growth arrest specific transcription factor 5) or miR-186-5p [75]. The former can upregulate the expression of apoptosis-related genes such as p53 and caspase3 in ECs, thereby promoting EC apoptosis [75]. The latter can promote the survival and migration of SMCs in AS by targeting SH2-containing inositol 5′-phosphatase (SHIP2) [75]. Furthermore, exosomes derived from ox-LDL-treated macrophages containing miRNA-146a can exacerbate AS by inducing oxidative stress and promoting the formation of neutrophil extracellular traps [76].

It is noteworthy that under the same stimulus, the same type of cells can secrete exosomes carrying different bioactive molecules. Whether these molecules have exosome-structural selectivity or receptor cell-targeting specificity remains unclear [77]. Additionally, under the stimulation of nicotine, macrophages can produce exosomes containing miRNA-21-3p, which promote VSMC proliferation and migration by reducing the expression of phosphatase and tensin homolog (PTEN), thereby promoting AS progression [78]. Nicotine can also induce monocytes to produce exosomes containing miR-155, which can induce EC dysfunction *via* the nuclear factor kappa-B (NF-κB) signaling pathway [79]. This indicates that different cells can produce exosomes with different cargoes under the same stimulus, but whether these exosomes have regulatory intersections is yet to be further explored.

Furthermore, some cells outside AS plaques can secrete exosomes that affect the lesions through the circulatory system, thereby influencing AS progression. A recent study suggested that sleep deprivation can promote AS progression by inducing EC inflammation *via* circulating exosomes carrying miRNA-182-5p [80]. In cases of obesity, visceral fat can secrete exosomes containing miRNA-27b-3p, which promote EC inflammation and AS progression by inhibiting peroxisome proliferator activated receptor α (PPARα) [81]. This implied that the progression of AS might be influenced by exosomes from various cells, and exploring the regulatory intersections of these exosomes and their effects on cell function could offer new opportunities for treating AS-related diseases. Moreover, exosomes can serve as biomarkers to some extent. For example, measuring plasma exosomal miRNA-150 levels can predict vascular inflammation and the occurrence of AS [82]. Assessing CD11b+ (macrophage maker)/CD66+ (neutrophil marker) microvesicle levels in the circulation of patients with unstable plaques can evaluate plaque vulnerability and identify high-risk individuals with asymptomatic plaque rupture [83]. However, challenges remain in terms of exosome stability, classification, isolation and purification, requiring further exploration and breakthroughs [84].

## 6. Other Pathological Mechanisms and AS

### 6.1 Endothelial-to-Mesenchymal Transition (EndMT) 

Recent studies have highlighted EndMT as a novel mechanism in AS development [85, 86], especially in conditions like premature aging. This process contributes to the formation of plaques by promoting endothelial cell transition to a mesenchymal state, leading to increased plaque instability.

### 6.2 TREM2 in Inflammation 

New research has identified TREM2 (triggering receptor expressed on myeloid cells 2) as a key regulator in macrophage behavior, affecting inflammation and plaque stability [87, 88]. Targeting TREM2 has shown promise in reducing inflammation and atherosclerotic plaque development.

### 6.3 MicroRNAs (miRNAs) 

The role of miRNAs in AS, particularly in regulating gene expression during plaque formation and progression, has gained attention [89]. These small RNA molecules influence various pathways involved in AS and could be considered both as therapeutic targets and biomarkers. For example, the manuscript does not mention inclisiran, a notable omission given its growing significance in lipid-lowering therapies. Inclisiran is a small interfering RNA (siRNA) that targets proprotein convertase subtilisin/kexin type 9 (PCSK9) and has demonstrated effective LDL-C reduction with long-lasting effects. Its biannual dosing regimen provides a major advantage over current therapies like PCSK9 inhibitors, potentially improving patient compliance and outcomes. Inclisiran has shown significant potential in recent clinical trials for both primary and secondary prevention of cardiovascular diseases [89].

## 7. New Lipid-Lowering Drugs for AS

CVDs are a leading cause of death worldwide, with AS being one of the major contributing factors [90, 91]. Elevated levels of LDL-C are an independent risk factor for atherosclerosis, and lowering LDL-C levels is a crucial strategy for primary and secondary prevention of atherosclerotic CVDs [92]. Statins remain the cornerstone therapy for lowering LDL-C, and large-scale randomized trials have demonstrated significant benefits of statin therapy in lipid-lowering treatment for high-risk patients [93]. However, due to the dose-dependent nature and severe side effects of statins, patients often discontinue the medication before reaching the lipid levels recommended by guidelines, necessitating the switch to alternative lipid-lowering drugs. Currently, several novel lipid-lowering drugs have entered clinical trials and applications, achieving breakthrough progress. Identifying new alternative lipid-lowering medications is of great importance for reducing the risk of atherosclerotic CVDs.

### 7.1 Bempedoic Acid

Bempedoic acid (ETC-1002) is a novel drug for lowering LDL-C by inhibiting ATP citrate lyase (ACL) which is a cytoplasmic enzyme that catalyzes the cleavage of mitochondrial-derived citrate into oxaloacetate and acetyl-CoA. The latter is a common substrate for the synthesis of cholesterol and fatty acids. In the liver, ETC-1002 can inhibit cholesterol synthesis and then induce a compensatory upregulation of LDL receptors [94] and promote hepatic uptake of LDL particles, thereby lowering blood LDL-C levels. Additionally, it can inhibit cholesterol and fatty acid synthesis pathways by targeting the rate-limiting enzymes 3-hydroxy-3-methylglutaryl-coenzyme A (HMG-CoA) reductase and acetyl-CoA carboxylase, respectively [95]. Among ACL inhibitors tested clinically, ETC-1002 is at the forefront of clinical development, which is an orally administered small molecule with a half-life of 15–24 hours and it is rapidly absorbed in the small intestine. Unlike statins, it does not compete for uptake pathways in the liver as it binds to different cell surface receptors. Therefore, current clinical trial results strongly suggest that ETC-1002 may be a new treatment option for lowering LDL-C.

To date, ETC-1002 has undergone several phase I and II clinical trials. Multiple phase II studies have also shown that ETC-1002 can reduce LDL-C levels in combination with statins or ezetimibe [96]. Notably, the combination of ETC-1002 and ezetimibe showed the greatest reduction in LDL-C in patients with a history of statin intolerance [96]. However, whether ETC-1002 has clinically relevant beneficial effects on other cardiometabolic risk factors (such as hyperglycemia and insulin resistance) remains unclear, requiring further clinical investigation.

### 7.2 PCSK9 Inhibitors

PCSK9 is primarily expressed and secreted by the liver [97] which is bound to LDL receptors in the cells inside and outside, promoting hepatic clearance of LDL-C and ultimately leading to elevated circulating LDL-C levels. Current PCSK9 inhibitors mainly include evolocumab, alirocumab and bococizumab. These antibodies can bind to the catalytic and prodomain regions of PCSK9, blocking its interaction with LDLR and neutralizing PCSK9 activity.

Existing evidence has shown that PCSK9 inhibition demonstrated benefits in key lipid metabolism components across various clinical settings. Current clinical trial results indicate that evolocumab can significantly reduce lipid levels in patients without hyperlipidemia, with sustained effects for up to 2.2 years [97]. Clinical studies measuring atherosclerotic volume percentage *via* ultrasound further supported the lipid-lowering effects of evolocumab. A study involving 968 patients undergoing coronary angiography revealed that evolocumab significantly reduced atherosclerotic volume compared to placebo, and this reduction was associated with decreased LDL-C levels during treatment [98]. Alirocumab was the first PCSK9 inhibitor approved by the US Food and Drug Administration (FDA) in July 2015 for adult patients with hypercholesterolemia who have not achieved adequate LDL-C reduction with maximally tolerated statin therapy or who require additional LDL-C lowering for clinical atherosclerotic CVDs [99]. Multiple clinical trials have found that alirocumab has similar lipid-lowering effects to evolocumab. While most studies focused on changes in cholesterol levels from baseline to week 24, the ODYSSEY LONG TERM trial, a large phase III clinical study, conducted across 27 countries including Africa, Europe, North America and South America, demonstrated that alirocumab continue to effectively lower LDL-C levels at 78 weeks [100]. Bococizumab, a humanized monoclonal antibody with approximately 3% murine sequence, showed high titers of anti-drug antibodies in most patients, significantly diminishing the extent and durability of LDL-C reduction. Moreover, in patients without anti-drug antibodies, the degree of LDL-C reduction varied greatly. As a result, the development of bococizumab was terminated in November 2016 [97].

Although a previous study has also reported localized reactions on injection site, myalgia and neurocognitive disturbances by using PCSK9 inhibitors, the lipid-lowering efficacy and potential clinical benefits derived from CVDs are notable [101]. FDA-approved PCSK9 inhibitors have confirmed that inhibiting PCSK9 can effectively reduce LDL-C levels.

### 7.3 Angiopoietin Like Protein 3 Inhibitor

Angiopoietin like protein 3 (ANGPTL3) can inhibit the activity of endothelial lipase and lipoprotein lipase (LPL), leading to elevated levels of LDL and triglycerides [102]. Studies have found that loss of ANGPTL3 function is associated with lipid reduction, which makes ANGPTL3 a highly attractive pharmacological target [102]. So far, two drugs have been confirmed to inhibit ANGPTL3. The first drug is called evinacumab, which lowers LDL-C and triglyceride levels by increasing the activity of LPL and other related metabolic enzymes. In patients with homozygous familial hypercholesterolemia, evinacumab can lower plasma LDL levels by 20% to 90% and triglyceride levels by 50% [103]. The second drug is ANGPTL3 specific antisense oligonucleotides (ASOs), which can inhibit the synthesis of ANGPTL3. Research has shown that in healthy individuals, ASOs can dose-dependently reduce triglyceride levels by 30% to 60%. Additionally, ASOs positively lower LDL and improve high density lipoprotein (HDL) levels [104].

### 7.4 Microsomal Triglyceride Transporter (MTP) Inhibitors

MTP facilitates the assembly and secretion of very-low-density lipoprotein (VLDL) particles by transferring triglycerides to ApoB. Lomitapide, an MTP inhibitor, has been approved for the treatment of familial hypercholesterolemia. Existing clinical studies have found that lomitapide not only can lower LDL-C levels but also plasma high density lipoprotein-cholesterol (HDL-C) and apolipoprotein AⅠ levels [105, 106]. Although clinical research indicated that lomitapide has a high incidence of gastrointestinal dysfunction, elevated transaminases and fatty liver, the adverse effects of these complications do not outweigh the benefits of reducing the risk of atherosclerotic CVDs. Nevertheless, due to the potential risk of these complications, lomitapide is currently used only as an adjunct therapy for patients with homozygous familial hypercholesterolemia.

### 7.5 Diacylglycerolyltransferase 1 Inhibitor 

Diacylglycerolyltransferase 1 (DGAT1) plays a critical role in lipid metabolism by catalyzing the triglyceride synthesis pathway. Pradigastat is an oral DGAT1 inhibitor. In previous Phase I and II clinical trials, 6 patients with familial chylomicronemia syndrome exhibited a dose-dependent reduction in triglyceride levels of 41% to 70% over a 21-day treatment period [107]. In another trial involving 106 overweight or obese patients, pradigastat was administered in multiple dose-escalation regimens. The results indicated that pradigastat could lower postprandial glucose, insulin and triglyceride levels, while increasing postprandial glucagon-like peptide-1 (GLP-1) levels [108]. The main gastrointestinal adverse effects included nausea and diarrhea, with the most pronounced effects observed at a 10 mg dose. A low-fat diet was found to improve tolerability, but gastrointestinal reactions still limited the continued development and widespread use of this drug.

### 7.6 Peroxisome Proliferator Activated Receptor α Agonist

PPARα agonists are a class of transcription regulators involved in modulating lipid metabolism and the expression of key apolipoproteins [109]. Pemafibrate, a novel and selective PPARα modulator, is designed to maximize the beneficial effects of currently used fibrates while minimizing adverse reactions. Both *in vivo* and *in vitro* experiments have demonstrated that pemafibrate has a stronger activating effect on PPARα compared to previous fibrates. A recent randomized clinical study indicated that pemafibrate is more effective than fenofibrate in reducing triglyceride levels and has a lower incidence of adverse reactions [110].

### 7.7 Acetyl CoA Carboxylase Inhibitor

Gemcabene is an Acetyl CoA carboxylase (ACC) inhibitor that reduces hepatic triglyceride and cholesterol production while enhancing the clearance of VLDL cholesterol. Previous phase II clinical trials have shown that gemcabene significantly lowers plasma levels of LDL-C, very low density lipoprotein-cholesterol (VLDL-C), apolipoprotein C-III and triglycerides compared to placebo. Currently, gemcabene is a potentially valuable treatment option as it can lower LDL-C independently of LDL receptor function for patients with homozygous familial hypercholesterolemia.

### 7.8 Antisense Oligonucleotides

Current research suggests that lipoprotein(a) (Lp(a)) particles may have adverse effects on cardiovascular health in multiple ways. The Lp(a) may stimulate platelet activation and aggregation, which contributes to thrombus formation and increases the risk of arterial occlusion and thrombosis. In addition, Lp(a) particles also promoted the abnormal proliferation of vascular smooth muscle cells, and increased the formation of foam cells, and eventually led to the formation of atherosclerotic plaques and necrosis of the center of the artery wall. Levels of Lp(a) are not significantly affected by lifestyle modifications or most lipid-lowering therapies, including statins. However, new therapies, such as ASOs targeting apolipoprotein(a) synthesis, have shown promising results in reducing Lp(a) levels and cardiovascular risk [111]. At present, there are over 57 ASOs entering the mid clinical stage and beyond globally. The clinical application market is relatively blank, with a broad development space in the future.

### 7.9 Other Lipid-Lowering Drugs for AS

Lecithin-cholesterol acyltransferase (LCAT) is a plasma enzyme that primarily catalyzes the esterification of cholesterol during the formation of HDL. ACP-501 is a recombinant human LCAT solution, and clinical trial results have shown that the use of ACP-501 is associated with a 42% increase for HDL-C levels and a 22% decrease for cholesterol esters in patients with atherosclerotic CVDs. Eicosapentaenoic acid ethyl ester has been approved for the treatment of severe hypertriglyceridemia, reducing triglyceride levels and significantly reducing the risk of cardiovascular events in patients with statin intolerance. Recent clinical trials have also demonstrated that patients treated with eicosapentaenoic acid ethyl ester experienced a reduction in cardiovascular events by approximately 25% compared to the control group, with a trend towards reduced plaque volume. Therefore, the addition of eicosapentaenoic acid ethyl ester may be a beneficial approach for lowering LDL-C levels in patients with elevated triglyceride levels.

## 8. Conclusions

AS is a chronic inflammatory condition characterized by plaque formation in the vascular wall, resulting from various factors. Extensive research in recent years has provided new insights and intervention strategies from multiple perspectives, yet significant breakthroughs in the mechanisms of AS and the treatment of the diseases have not been achieved. AS-related conditions remain a leading cause of mortality worldwide. Among the many factors contributing to AS, inflammation remains central, but the efficacy of anti-inflammatory treatments has been suboptimal, highlighting the complexity of AS pathogenesis and the heterogeneity of outcomes under different influences. Currently, lipid-lowering therapy for atherosclerotic CVDs still relies primarily on statins due to their well-established clinical efficacy and long-term use. However, due to their dose-dependent nature and significant adverse effects, alternative lipid-lowering therapies become necessary when patients reach the maximum recommended dose of statins or experience severe adverse reactions. Novel lipid-lowering drugs have entered development and clinical trial phases, with several methods demonstrating significant efficacy and good safety and tolerability, whether used as monotherapy or in combination with statins or ezetimibe. Therefore, the development and application of novel lipid-lowering drugs remain crucial for the prevention and treatment of CVDs. The good news is that more and more research has focused on new mechanisms of AS formation involved in gut microbiota, pyroptosis, ferroptosis, autophagy, cuproptosis, exosomes and non-coding RNA [6, 7, 8, 9, 10], which might provide theoretical basis and potential drug therapeutic targets for the prevention and treatment of AS in future.
